# Hidden Glutathione Transferases in the Human Genome

**DOI:** 10.3390/biom13081240

**Published:** 2023-08-12

**Authors:** Aaron J. Oakley

**Affiliations:** School of Chemistry and Molecular Bioscience, Faculty of Science, Medicine and Health, University of Wollongong, Wollongong, NSW 2522, Australia; aarono@uow.edu.au; Tel.: +61-2-4221-4347

**Keywords:** glutathione transferase, intracellular chloride channel, metaxin, failed axon connections homolog, ganglioside-induced differentiation-associated protein, glutathione S-transferase C-terminal domain-containing protein, multi-tRNA synthetase complex, eukaryotic elongation factor 1, structure prediction

## Abstract

With the development of accurate protein structure prediction algorithms, artificial intelligence (AI) has emerged as a powerful tool in the field of structural biology. AI-based algorithms have been used to analyze large amounts of protein sequence data including the human proteome, complementing experimental structure data found in resources such as the Protein Data Bank. The EBI AlphaFold Protein Structure Database (for example) contains over 230 million structures. In this study, these data have been analyzed to find all human proteins containing (or predicted to contain) the cytosolic glutathione transferase (cGST) fold. A total of 39 proteins were found, including the alpha-, mu-, pi-, sigma-, zeta- and omega-class GSTs, intracellular chloride channels, metaxins, multisynthetase complex components, elongation factor 1 complex components and others. Three broad themes emerge: cGST domains as enzymes, as chloride ion channels and as protein–protein interaction mediators. As the majority of cGSTs are dimers, the AI-based structure prediction algorithm AlphaFold-multimer was used to predict structures of all pairwise combinations of these cGST domains. Potential homo- and heterodimers are described. Experimental biochemical and structure data is used to highlight the strengths and limitations of AI-predicted structures.

## 1. Introduction

Since glutathione transferase (GST) activity was discovered in rat liver and was postulated to play a role in drug detoxification [1], decades of research has led to the identification and isolation of multiple classes of GST from bacteria to man with a considerable array of catalytic and binding activities. In 1991, the first glutathione transferase structure was determined [2,3], the pi-class isozyme from pig in complex with glutathione-sulfonic acid (PDB ID 2GSR). Several features of that structure are now known to be typical of the cytosolic GSTs (cGSTs): it is a dimer, and each monomer contains an N-terminal domain (NDT) (having the thioredoxin-like βαβαββα topology) and a unique C-terminal domain (CTD) composed of α-helixes. Many crystal structures of cGSTs have revealed the binding location of glutathione (GSH) in the N-terminal domain (the “G-site”) and the adjacent binding site for (often hydrophobic) co-substrates (the “H-site”). While hundreds of GST structures have been reported in organisms ranging from bacteria to man, the set of human proteins known to adopt the cGST fold can be regarded as incomplete. However, recent advances in protein structure prediction provide the tools to discover and analyze these “hidden” GSTs. Not considered here are the microsomal GSTs, which are trimeric, integral membrane proteins, and the mitochondrial kappa-class GST that form a distinct family of thioredoxin-fold-containing proteins [4].

With the availability of deep learning algorithms such as AlphaFold [5] and RoseTTAfold [6], we now have tools to make reliable predictions of protein structures. Briefly, these artificial intelligence (AI) systems use a neural network to extract the relationship between a protein’s sequence and its 3D structure based on existing experimental data. Employed for the bulk of this study is AlphaFold, which uses a three-stage process to predict protein structures. In the first stage, a multiple sequence alignment (MSA) is generated for the target protein(s). In the second stage, the “evoformer block”, consisting of a series of interconnected layers, processes the input amino acid sequence using an “attention mechanism” that captures evolutionary information from related protein sequences. This mechanism allows the network to selectively focus on different parts of the sequence based on their importance for predicting the protein’s structure. Information from the evoformer feeds into the third stage, the “structure module”, which creates an explicit 3D structure. In a typical prediction run, the information from the structure prediction module is recycled three times through the evoformer and structure modules. Along with 3D models, AlphaFold provides a per-residue confidence metric called predicted local distance difference test (pLDDT) on the interval [0, 1]. A higher pLDDT implies higher confidence. In predicted structures, well-structured regions typically have high pLDDT scores. Loops and regions near termini (often missing in crystal structures) tend to have low pLDDT scores.

As of this writing, the EBI AlphaFold database contains over 230 million predictions of 3D protein structures from hundreds of organisms including humans. In this study, a structure-based search of this database was used to reveal all human proteins predicted to contain the cGST fold. A limitation of this database is that it contains only monomeric structures. Since many classes of GST-domain-containing proteins form dimers, I used AlphaFold-multimer [7] to predict complexes of all pairwise combinations of human GST-domain-containing proteins to reproduce known and predict new homo- and heterodimer structures. The quality of the predictions were assessed using metrics that AlphaFold generates for its models and using published structural and biochemical data. Insights gained include possible functions of poorly understood proteins and proposals for new protein–protein interactions involving cGST-containing domains.

## 2. Materials and Methods

Crystal structures of human proteins containing the cytosolic GST fold were obtained from the Protein Data Bank (PDB) and were used in DALI [8] searches through the set of human protein structures in the European Bioinformatics Institute AlphaFold database (EBI, Cambridge, UK). Redundant structures were removed.

Using amino acid sequences corresponding to the cGST domains in the proteins identified above, AlphaFold-multimer (ColabFold distribution, version 1.5.0; [9]) was used to predict the structures of all pairwise domain combinations. Within ColabFold, the “alphafold2_multimer_v3” model was used, which uses weights derived from training on PDB structures deposited up to September 30, 2021. For each prediction, five models were generated, and the top-ranked prediction was selected for further analysis. Assessment of model quality was based on AlphaFold’s intrinsic model accuracy estimate: predicted template modeling-score (pTM). AlphaFold-multimer gives a modified score for interactions between residues of different chains: interface pTM (ipTM). To assess the quality of predictions, model confidence (MC) was calculated “= 0.8 × ipTM + 0.2 × pTM” [7].

ChimeraX [10] was used for structure alignment and figure production. Clustal Omega [11] was used to generate a phylogram based on the GST-domain sequences.

## 3. Results

**Figure 1:**
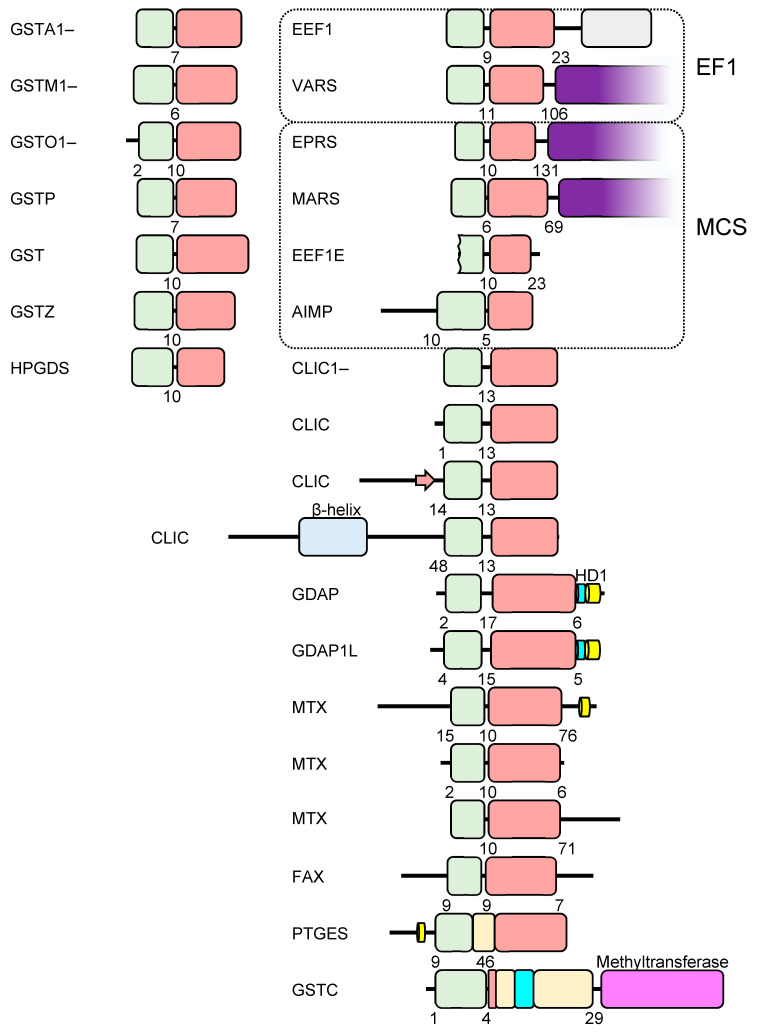
Domain organization of cGSTs in the human genome. The thioredoxin and C-terminal domains are indicated in pale green and pink, respectively. Numbers indicate the length of sequence before, between and after the NTDs and CTDs. Putative trans-membrane helices are indicated in yellow. tRNA synthetase domains are indicated in purple. Additional structure elements are described in the text.

Searches of the EBI AlphaFold database revealed a total of 39 human proteins predicted to contain the cGST fold (Table 1) that will be briefly described here. The domain organization of all hits are shown in Figure 1. The structures of the domains colored by pLDDT value are shown in Figure S1 and a phylogram based on the sequences is shown in Figure S2.

Slightly less than half of the proteins identified fall into previously identified classes of GST, including the alpha, mu, pi, theta, zeta and omega classes (18 proteins). The alpha-, mu- and pi-class GSTs are common in mammalian genomes and are well known for their role in phase II detoxification. They form a distinct clade in the phylogram (Figure S2). Most characterized reactions involve GSH-conjugating activity with electrophiles. Some GSTs have additional roles. For example, the human pi-class GST modulates the activities of the mitogen-activated protein kinase (MAPK) signaling pathway via direct interactions with apoptosis signal-regulating kinase (ASK1) [12] and c-Jun N-terminal kinase 1 (JNK1) [13].

The hematopoietic prostaglandin D synthase (HPGDS), also known as sigma-class GST (GSTS1), was identified. HPGDS catalyzes the GSH-dependent conversion of prostaglandin H_2_ (PGH_2_) to prostaglandin D_2_ (PGD_2_) [14].

Prostaglandin E Synthase 2 (PTGES2), also known as microsomal prostaglandin E synthase type 2, was found. It catalyzes conversion of PGH_2_ to prostaglandin E_2_ (PGE_2_) [15]. In addition to cGST-like motifs, this protein contains an insertion between the N- and C-terminal domains that results in an unusual dimerization interaction.

Three proteins classified as metaxins (MTX1, MTX2 and MTX3), which are associated with mitochondrial import and trafficking [16], were found.

Six intracellular chloride channels (CLIC1 to CLIC6) were identified. The CLICs pose an interesting challenge for structure prediction algorithms. While most experimental CLIC protein structures are characterized by the cGST fold, CLICs have both soluble and integral membrane forms [17]. Furthermore, CLIC1 has been shown to adopt two distinct soluble conformations [18].

Several hits correspond to protein domains involved in the organization of tRNA synthetase. These include eukaryotic translation elongation factor 1 epsilon 1 (EEF1E1), Aminoacyl tRNA synthetase complex interacting multifunctional protein 2 (AIMP2), Glutamyl-prolyl-tRNA synthetase 1 (EPRS1) and Methionyl-tRNA synthetase 1 (MARS1), which are components of the multi-tRNA synthetase complex (MSC) [19]. Eukaryotic translation elongation factor 1 gamma (EEF1G) and Valyl-tRNA synthetase 1 (VARS1) are components of the eukaryotic elongation factor 1 (eEF1) complex [20].

The remaining hits are relatively poorly characterized proteins: ganglioside-induced differentiation-associated protein 1 (GADP1), ganglioside-induced differentiation-associated protein-1-like 1 (GADP1L1), failed axon connections homolog (FAXC) and glutathione S-transferase C-terminal domain-containing protein (GSTCD).

**Table 1:** Glutathione-transferase-fold-containing proteins in the human genome.

Gene ^1^	Location	Name	Comment ^2^
*GSTA1*	6p12.2	Glutathione S-transferase alpha 1	Substrates: Δ^5^AD, BCDE, BPDE, Busulfan, Chlorambucil, DBADE, DBPDE, BPhDE, *N*-a-PhIP
*GSTA2*	6p12.2	Glutathione S-transferase alpha 2	CuOOH, DBPDE, 7-chloro-4-nitrobenz-2-oxa-1,3-diazole
*GSTA3*	6p12.2	Glutathione S-transferase alpha 3	Substrates: Δ^5^AD, Δ^5^-pregnene-3,20-dione, DBPDE
*GSTA4*	6p12.2	Glutathione S-transferase alpha 4	Substrates: COMC-6, EA, 4-hydroxynonenal, 4-hydroxydecenal
*GSTA5*	6p12.2	Glutathione S-transferase alpha 5	Uncharacterized
*GSTM1*	1p13.3	Glutathione S-transferase mu 1	Substrates: *trans*-4-phenyl-3-buten-2-one, BPDE, CDE, DBADE, *trans*-stilbene oxide, styrene-7,8-oxide
*GSTM2*	1p13.3	Glutathione S-transferase mu 2	Substrates: COMC-6, 1,2-dichloro-4-nitrobenzene, aminochrome, dopa *O*-quinone, PGH_2_ → PGE_2_
*GSTM3*	1p13.3	Glutathione S-transferase mu 3	Substrates: BCNU, PGH_2_ → PGE_2_
*GSTM4*	1p13.3	Glutathione S-transferase mu 4	CDNB
*GSTM5*	1p13.3	Glutathione S-transferase mu 5	Uncharacterized
*GSTO1*	10q25.1	Glutathione S-transferase omega 1	Substrates: MMA, dehydroascorbate, S-(4-nitrophenacyl)glutathione
*GSTO2*	10q25.1	Glutathione S-transferase omega 2	Glutaredoxin-like activities
*GSTP1*	11q13.2	Glutathione S-transferase pi 1	Substrates: CDNB, acrolein, base propenals, BPDE, CDE, Chlorambucil, COMC-6, EA, Thiotepa
*GSTT1*	22q11.23	Glutathione S-transferase theta 1	A pseudogene in the reference genome. Protein coding in some individuals. Substrates: BCNU, butadiene epoxide, CH_2_Cl_2_, EPNP, ethylene oxide
*GSTT2*	22q11.23	Glutathione S-transferase theta 2	CuOOH, 1-menaphthyl sulfate
*GSTT2B*	22q11.23	Glutathione S-transferase theta 2B	A pseudogene in some individuals. Substrates: CuOOH, 1-menaphthyl sulfate
*GSTT4*	22q11.23	Glutathione S-transferase theta 4	Uncharacterized
*GSTZ1*	14q24.3	Maleylacetoacetate isomerase	Substrates: dichloroacetate, fluoroacetate, 2-chloropropionate, malelyacetoacetate
*HPGDS*	4q22.3	Hematopoietic prostaglandin D synthase	PGH_2_ → PGD_2_
*PTGES2*	9q34.11	Prostaglandin E synthase 2	PGH_2_ → PGE_2_
*GDAP1*	8q21.11	Ganglioside-induced differentiation-associated protein 1	Mitochondrial transport
*GDAP1L1*	20q13.12	Ganglioside-induced differentiation-associated protein-1-like 1	Mitochondrial transport
*CLIC1*	6p21.33	Chloride intracellular channel 1	Intracellular chloride ion channel
*CLIC2*	Xq28	Chloride intracellular channel 2	Intracellular chloride ion channel
*CLIC3*	9q34.3	Chloride intracellular channel 3	Intracellular chloride ion channel
*CLIC4*	1p36.11	Chloride intracellular channel 4	Intracellular chloride ion channel
*CLIC5*	6p21.1	Chloride intracellular channel 5	Intracellular chloride ion channel
*CLIC6*	21q22.12	Chloride intracellular channel 6	Intracellular chloride ion channel
*MTX1*	1q22	Metaxin 1	Mitochondrial outer membrane component
*MTX2*	2q31.1	Metaxin 2	Mitochondrial outer membrane component
*MTX3*	5q14.1	Metaxin 3	Mitochondrial outer membrane component
*FAXC*	6q16.2	Failed axon connections homolog	
*GSTCD*	4q24	Glutathione *S*-transferase C-terminal domain-containing protein	Probable methyltransferase
*EEF1E1*	6p24.3	Eukaryotic translation elongation factor 1 epsilon 1	MSC component
*AIMP2*	7p22.1	Aminoacyl tRNA synthetase complex interacting multifunctional protein 2	MSC component
*EPRS1*	1q41	Glutamyl-prolyl-tRNA synthetase 1	MSC component
*MARS1*	12q13.3	Methionyl-tRNA synthetase 1	MSC component
*EEF1G*	11q12.3	Eukaryotic translation elongation factor 1 gamma	eEF1 component
*VARS1*	6p21.33	Valyl-tRNA synthetase 1	eEF1 component

^1^ HUGO Gene Nomenclature Committee recommended names used. ^2^ Abbreviations: Δ^5^AD, Δ^5^-androstene-3,17-dione; BCDE, benzo[*g*]chrysene diol epoxide; BCNU, 1,3-bis(2-chloroethyl)-1-nitrosourea; BPDE, benzo[*a*]pyrene diol epoxide; BPhDE, benzo[*c*]phenanthrene diol epoxide; CDE, chrysene1,2-diol 3,4-epoxide; CDNB, 1-chloro-2,4-dinitrobenzene; COMC-6, crotonyloxymethyl-2-cyclohexenone; DBADE, dibenz[*a*,*h*]anthracene diol epoxide; DBPDE, dibenzo[*a*,*l*]pyrene diol epoxide; EA, ethacrynic acid; EPNP, 1,2-epoxy-3-(*p*-nitrophenoxy)propane; *N*-a-PhIP, *N*-acetoxy-2-amino-1-methyl-6-phenylimidazo[4,5-*b*]pyridine; MMA, mono-methylarsonic acid; MSC, Multi-tRNA synthetase complex. Substrate data from [21] and elsewhere.

RMSD-based comparisons show that the predicted GST structures are very similar to high-resolution crystal structures of human wild-type proteins (where they are available) (Table 2). In general, the greatest deviations occur in surface loops or near the termini. In general, these regions are poorly ordered in crystal structures and show lower pLDDT scores in predictions (Figure S1). Specific cases will be detailed below.

### 3.1. Prediction of GST Homo- and HeteroDimers

**Figure 2:**
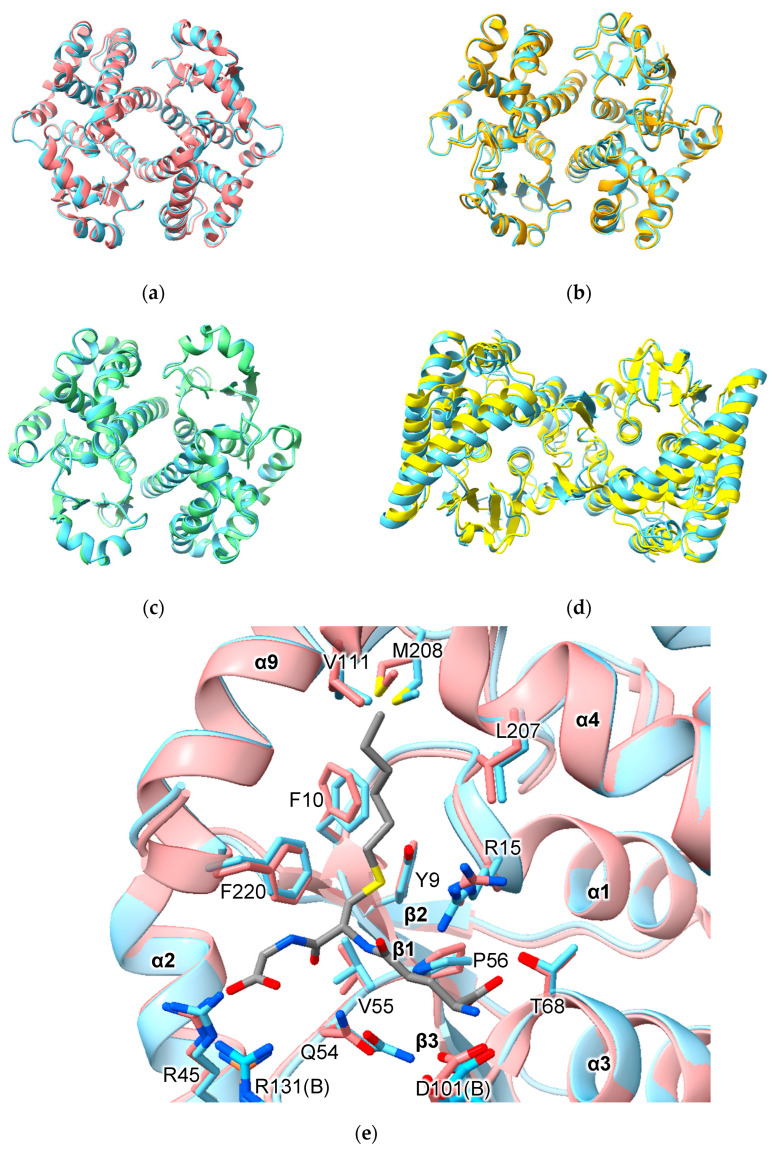
Comparison of predicted human versus experimentally determined structures of GST dimers. (**a**) human GSTA1-1 (PDB ID 1K3Y); (**b**) human GSTM1-1 (PDB ID7 BEU); (**c**). human GSTP1-1 (PDB ID 5J41); (**d**) *Macaca fascicularis* PTGES2 (PDB ID 1Z9H); (**e**) active site of GSTA1-1 with S-hexyl glutathione bound. In all cases, the predicted structure is indicated in sky blue.

AlphFold predicted homodimers of all human alpha-, mu-, pi-, theta- and omega-class GSTs and HPGDS are consistent with crystallographic results obtained to date. For example, the homodimers GSTA1-1, GSTM1-1 and GSTP1-1 superimpose with RMSD values of 0.64 Å (over 422 Cα atoms), 0.58 Å (over 434 Cα atoms) and 0.35 Å (416 Cα atoms), respectively (Figure 2a–c). Of note is PTGES2, which has an unusual mode of dimerization thanks to a 46-residue insertion between the N- and C-terminal domains (Figure 1). Nevertheless, AlphaFold predicted the structure of this insertion and the dimerization interaction correctly (RMSD 1.92 Å over 548 Cα atoms) (Figure 2d). These RMSD values are similar to those obtained when comparing crystal structures of the same proteins.

There are relatively few biochemical studies of GST heterodimers. Within the alpha, mu- and theta-class GSTs, all isoforms are predicted, based on model confidence scores, to form heterodimers (Table S1). Data concerning the human alpha-class GSTs provide valuable context for the interpretation of predicted heterodimer models. Within the human alpha-class GSTs, the A4 isozyme, a phylogenetic outlier (Figure S2), has residues in its interface that distinguish it from the other alpha-class isozymes. Co-expression of human GSTA4 and GSTA1 in *E. coli* shows that, while each subunit prefers to form homodimers, it is possible to form the GSTA1-4 heterodimer. While both subunits were active with substrates CDNB, HNE or Δ^5^AD in the heterodimer, the specific activities and *k*_cat_ values were lower than the average values of the parent isozymes [22]. By contrast, the catalytic efficiencies (*k*_cat_/*K*_M_) of various heterodimers of rat alpha-class GSTs toward CDNB were predictable from the activities of their corresponding homodimers [23]. The model confidence for the predicted GSTA1-4 heterodimer structure is the same as for the GSTA1-1 homodimer (0.94). In fact, the model confidence for the GSTA1-4 heterodimer is higher than that for the GSTA4-4 homodimer (0.92). These data show that that model confidence scores should not be used as a proxy for multimerization preference or activity. While heterodimer formation is possible, and structures of heterodimers predicted with high confidence, they may not be preferred in vitro or in vivo.

An important aspect of structure prediction is the fidelity with which the algorithm reproduces the conformation of binding-site residues. Comparison of the active site of predicted and crystal structures of GSTA1-1 indicates that some side-chains show small differences in conformation. Of 14 residues in the active site, different rotamers were assigned to three residues in the model versus the crystal structure (Figure 2e). Residue R13 is assigned the **mtt**-180° rotamer in the model but adopts the **mtt**-85° rotamer in the crystal structure. Residue Q54 is assigned the **pt**-20° rotamer in the model but adopts the **mt**-30° conformation in the crystal structures. Finally, residue M208 is assigned the **mtm** rotamer in the model but adopts the **ttp** rotamer in the crystal structure. The consequence of these differences is that the placement of side-chains is similar but not identical. It is important to note that, while AlphaFold does not build ligands into predicted structures, the effects of ligand binding nonetheless may be imprinted on the structures on which the AI was trained. Thus, while the C-terminal helix (α9) is disordered in the unliganded structures (e.g., PDB 1GSD), it is nonetheless present in the predicted structures in a conformation observed in ligand-bound GSTA1-1 structures.

Few crystal structures of cGST domain-based heterodimers have been deposited in the PDB. However, published examples offer the opportunity to assess the quality of AlphaFold predictions of such assemblies. Comparison of the human GSTM2-M3 crystal structure solved at 2.8 Å resolution (PDB 3GTU) with the predicted structure yielded a RMSD of 0.69 Å over 434 Cα atoms. Comparison of the 2.6 Å crystal structure of the heterodimer of GST-like domains from EPRS1 and AIMP2 (PDB 5A34) with the AlphaFold prediction yielded an RMSD of 0.71 Å over 349 Cα atoms. The crystal structure of the GST-like domains of MARS1 and EEF1E1 (PDB 4BL7) with the predicted structure gave an RMSD of 0.80 Å over 366 Cα atoms.

**Figure 3:**
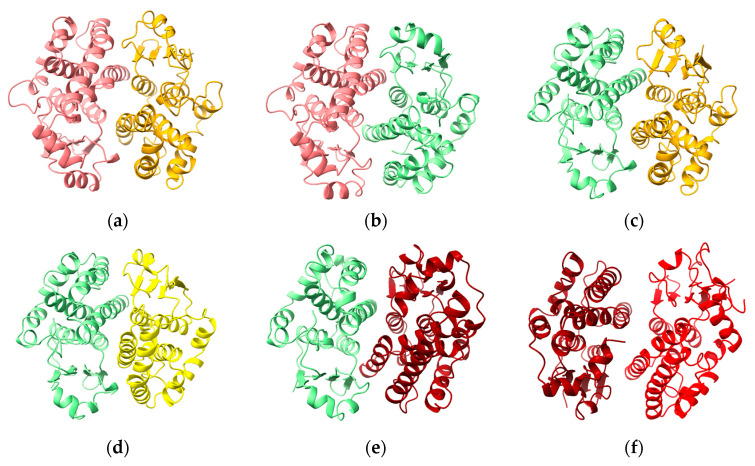
Predicted structures of heterodimers containing cGST domains: (**a**) GSTA1-M1; (**b**) GSTA1-P1; (**c**) GSTP1-M1; (**d**) GSTP1-HPGDS; (**e**) GSTP1-T1; (**f**) GSTT1-Z1.

A remarkable result is the prediction, with high confidence, of heterodimers of alpha-, mu-, pi- and theta-class—as well as other classes of—GST. (Selected heterodimers are shown in Figure 3.) While heterodimers within classes of GST are well known, there are few reports of heterodimers between classes of GST. Heterodimers of mu- and pi-class GSTs have been observed by incubation of rat GSTM2 and pig GSTP1 enzymes in phosphate buffer at 4 °C for 24 h [24]. The same researchers were unable to detect heterodimers of rat GSTs A1, A2 or A3 with the pi-class GST. However, the dissociation kinetics under experimental conditions may have been too slow to allow detection.

### 3.2. Metaxins and FAXC

Metaxin (from the Greek μεταξύ; “between”) was first identified in mice as essential for embryonic development [25] and later shown to be a mitochondrial outer membrane component [26]. In humans, three metaxins have been described (MTX1, MTX2 and MTX3). These proteins have been identified as components of the mitochondrial intermembrane space bridging (MIB) complex that includes SAM50 (also a component of the Sorting and Assembly Machinery or SAM complex), DNAJC11 and several other components [27]. Mandibuloacral dysplasia associated with MTX2 (MADaM) is a sever condition with symptoms including growth retardation and arises from homozygous null mutations in the human *MTX2* gene [28].

**Figure 4:**
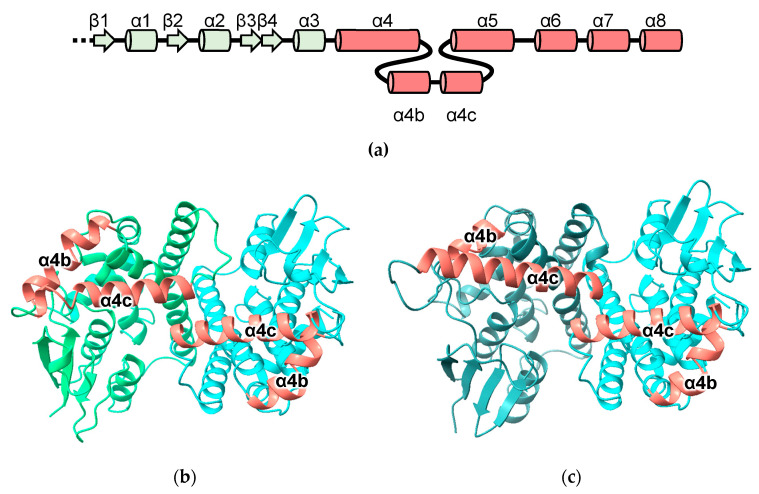
Predicted metaxin and FAXC structures: (**a**) topology diagram of metaxins and FAXC indicating helices inserted into the cGST domain (α4b and α4c); (**b**) MTX1-MTX2 (**c**) FAXC-MTX2.

The GST-like domains of the metaxins are predicted to incorporate helices inserted between the canonical α4 and α5 helices cover the “G-site”, which in these models is blocked and would be unable to bind GSH (Figure 4). In line with earlier predictions that MTX1 has a transmembrane domain near its C-terminus (Figure 1; residues 421 to 443) that is predicted to play a role in apoptosis [29], a hydrophobic helix is predicted for this region. The pattern of pairwise dimer predictions involving MTX2 and its close relatives suggests that MTX2 acts as a key interaction domain. The structures with the highest MC scores are MTX2 with MTX1, MTX3 and FAXC. Predicted heterodimers of MTX1-MTX2 and FAXC-MTX2 are shown in Figure 4b,c, respectively. Curiously, the MTX2 homodimer appears to be disfavored (MC = 0.28) and is predicted to form heterodimers with MTX1, MTX3 and FAXC, but not to form homodimers (Table S1).

There is little experimental or clinical data concerning FAXC (“Failed Axon Connections Homolog”). Transcriptomics reveal high levels of expression in the brain [30]. One report describes patients with developmental delay due to a 6q16.1 deletion that included the FAXC gene [31]. The human protein with the highest sequence similarity to FAXC is MTX2, with which FAXC is predicted to form a homodimer (MC = 0.90). Like MTX1 and MTX3, AlphaFold predictions of FAXC with other GST domains produced low ipTM scores with the exception of MTX2 (MC = 0.90). These data and phylogenetic analysis (Figure S2) suggest that FAXC is an outlying member of the metaxin family and, like MTX1 and MTX3, may form a heterodimer with MTX2.

### 3.3. GDAP1 and GDAP1L1

Ganglioside-Induced Differentiation-Associated Protein 1 (GDAP1) is a mitochondrial outer membrane protein involved in mitochondrial fission. GDAP1 mutations are associated with the autosomal recessive neurological disorder Charcot–Marie–Tooth disease type 4A. Mutations in GDAP1 that are associated with disease (mostly missense mutations) impede mitochondrial dynamics. Crystal structures of the mouse [32] and human [33] GDAP1 homologs confirmed the presence of the cGST fold. Efforts to detect the GSH-conjugating activity of GDAP have yielded mixed results, with some groups detecting no activity [32,34], and others detecting GSH-conjugating activity with EA, *p*-nitrobenzylchloride and EPNP [35]. GDAP1L1 is a paralogue of GDAP1 (59% sequence identity) and appears to function in mitochondrial fission. Expression of GDAP1L1 in macrophages is implicated in T-cell- and dendritic-cell-driven skin inflammation disease [36]. GDAP1 and GDAP1L1 appear to be distant relatives of the CLICs (Figure S2).

**Figure 5:**
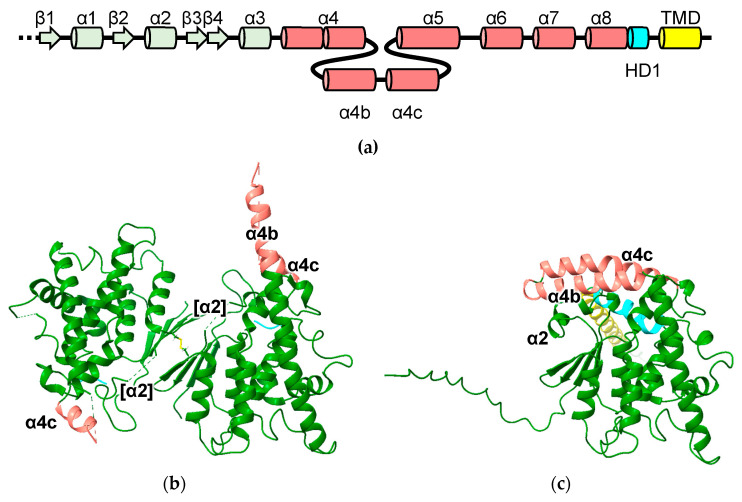
Experimental and predicted GDAP1 structures: (**a**) topology diagram of GDAP1 and GDAP1L1 indicating helices inserted into the cGST domain (α4b and α4c), HD1 and TMD domains; (**b**) Crystal structure of human GDAP1 residues 23 to 302 (PDB ID 7ALM) with the C88 disulfide in stick representation; (**c**) AlphaFold model of human GDAP1. Regions corresponding to inserted sequence (designated helices α4b and α4c in (**a**) are highlighted in pink. The HD1 domain (truncated in the crystal structure) is highlighted in cyan and the TMD domain in yellow. The region corresponding to helix α2 disordered in the crystal structure is indicated as [α2].

In addition to the cGST domain, GDAP has a sequence inserted between regions corresponding to helix α4 and α5, and an auto-inhibitory hydrophobic domain (HD1) followed by a trans-membrane domain (TMD) near its C-terminus (Figure 1 and Figure 5a). Mitochondrial fission and GST activity are dependent on HD1. Huber and co-workers proposed that HD1 switches between an autoinhibited mode, where HD1 blocks the catalytic site, and an active mode, where HD1 dissociates from the catalytic site and associates with the membrane [35]. HD1 is in a position to influence the position of the loop between strand β1 and helix α1. The GDAP1L1 sequence also features the HD1 and TMD motifs.

Crystal structures of mouse and human GDAP1 omit extensions at the N- and C-termini and are dimers with an atypical arrangement: the beta-sheets form a sandwich stabilized by an inter-chain disulfide bond between C88 of each monomer (Figure 5b). None of the GDAP1 crystal structures reported to date appear be competent at binding GSH due to helix α2 and flanking regions being disordered (Figure 5b) and residue P78 adopting a *trans* conformation. The equivalent proline in catalytically active cGSTs is in the *cis* conformation, is essential for GSH binding and is the only completely conserved G-site residue in the cGSTs. Intriguingly, the AlphaFold model of GDAP1 adopts a classic cGST-like structure including *cis*-proline in this region compatible with GSH binding (Figure 5c). Another key difference between model and crystal structures is the insertion between helices α4 and α5 (residues 154 to 200 in GDAP). In the AlphaFold model, this region consists of two helices forming a lid over the active site that contacts helix α2 like that seen in the predicted metaxin structures. This region is mostly disordered in crystal structures (Figure 5b). However, the residues that are present do not align with the AlphaFold model, leading to a higher RMSD (7.511 Å over 259 residues) compared to other models for which experimental structures are available (Table 2).

### 3.4. CLICs

Paradoxically, CLICs were first identified as chloride ion channels, yet they have a soluble form that adopts the cGST fold [37]. Several CLICs have been reported to spontaneously integrate into lipid bilayers. In addition to overall topology, CLICs contain features conserved in GSTs including the G-site *cis*-proline. They contain a conserved motif located between strand β1 and helix α1 that is also observed in glutaredoxins: CP(F/Y)C. A similar motif is also seen on the Omega-class GSTs (CPFA and CPYS in human GSTO1 and O2, respectively).

**Figure 6:**
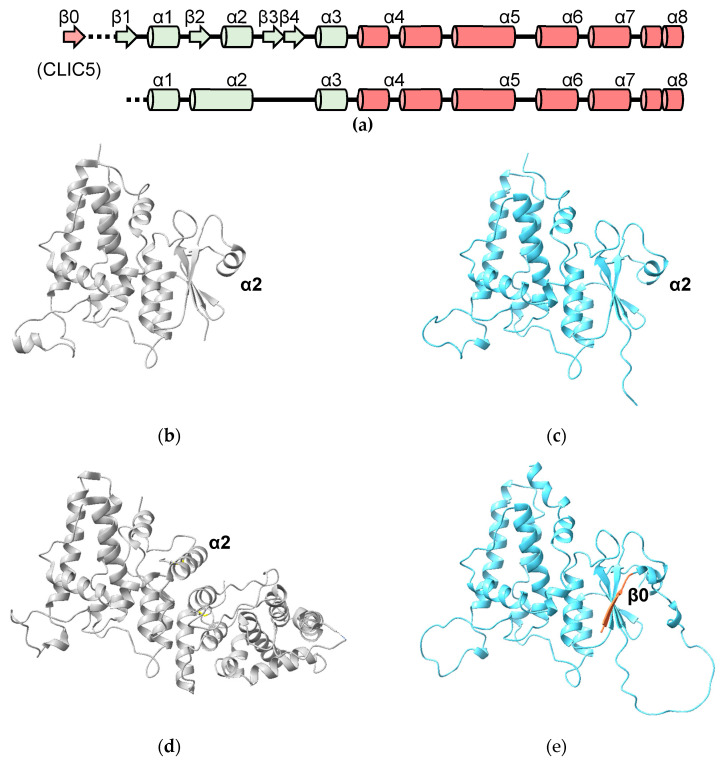
Experimental and predicted CLIC structures: (**a**) topology diagram of CLICs. The additional β-strand (“β0”) is found in CLIC5. Below, the topology of oxidized CLIC1 is shown; (**b**) crystal structure of human CLIC1 (PDB ID 1K0M); (**c**) AlphaFold model of human CLIC1; (**d**) crystal structure of oxidized CLIC1 (PDB ID 1RK4); (**e**) predicted structure of CLIC5 (residue 141–410) with additional strand β0 highlighted in pink.

The predicted structures of the human CLICs all have the cGST fold and are in excellent agreement with experimentally determined structures (where available) (Table 2). A noteworthy exception is the oxidized form of CLIC1, represented by PDB structure 1RK4. This contains an intramolecular disulfide between residues C24 and C59 and the N-terminal domain is rearranged [18]. The predicted model of CLIC1 matches the reduced, cGST-like form of CLIC1 (RMSD = 2.18 Å over 236 Cα atoms) and not the exceptional oxidized structure (RMSD = 7.47 Å over 213 Cα atoms) (Figure 6d).

**Figure 7:**
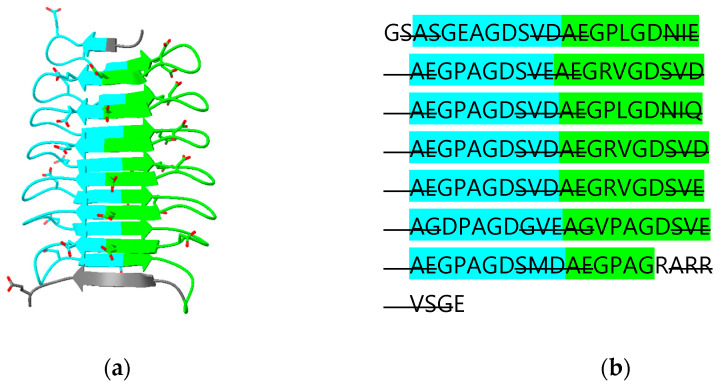
Predicted β-helix domain (residues 150-295) of human CLIC6: (**a**) cartoon diagram with acidic residues in stick form; (**b**) amino acid sequence with odd- and even-numbered repeats highlighted in cyan and green, respectively. β-strand regions are indicated with strikethrough.

CLIC 4, 5 and 6 have N-terminal extensions predicted to be largely disordered. The N-terminal extension on CLIC5 is predicted to contain an additional β-strand “β0” that runs parallel to strand β2 (Figure 6a,e). The N-terminal extension is deleted in the crystal structure of human CLIC5 (PDB ID 6Y2H) which, thus, does not contain this additional β-strand. The longest N-terminal extension (487 residues) in CLIC6 includes 14 copies of a decapeptide motif (consensus sequence AEGPAGDSVD; residues 150 to 295) [38]. This repeat region is predicted to form a right-handed, 15-stranded β-helix. The relationship between the secondary structure of the beta helix and the repeat is illustrated in Figure 7. The function of this domain is unknown. A DALI search of the PDB using this domain as a search model reveals an ice-binding protein from perennial ryegrass, *Lolium perenne* (PDB 3ULT), adhesin UspA1 from the Gram-negative bacterium *Moraxella catarrhalis* (PDB 3PR7) and tailspike protein TSP3 from bacteriophage CBA120 (PDB 6NW9).

In keeping with their known monomeric structures, no human CLIC is predicted to form dimers (Table S1). Predictions of heterodimers of CLIC GST domains with other GST-domain proteins produced negative results except with AIMP2. In a study investigating the role of CLIC4 in pancreatic β-cell apoptosis, mass spectrometry experiments demonstrated an interaction between CLIC4 and AIMP2 [39].

### 3.5. GSTCD

GSTCD has been implicated in the development of Chronic Obstructive Pulmonary Disease. GSTCD^−/−^ mice showed an increased lung TNF production in response to lipopolysaccharide. It was predicted to contain a methyltransferase domain [40].

**Figure 8:**
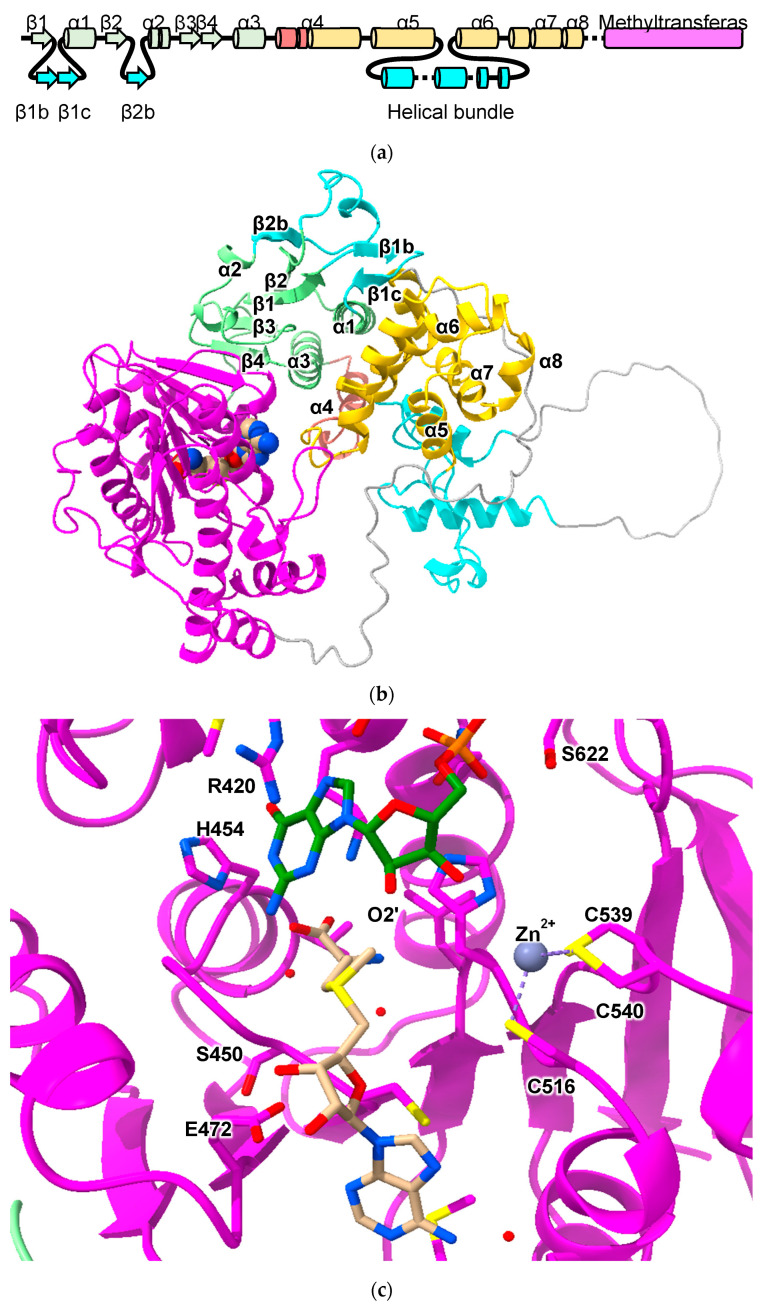
Predicted structure of GSTCD: (**a**) topology diagram of GSTCD indicating strands and helices inserted into the cGST domain (β1b, β1c and β2b). The component of the cGST CTD that is offset from its usual position is colored yellow. The methyltransferase domain is colored purple. (**b**) Predicted structure of GSTCD with the same color scheme in cartoon representation. SAM atoms are shown as spheres. (**c**) Cartoon diagram of GSTCD model with SAM (tan carbon atoms), Zn^2+^ and substrate RNA (green carbon atoms). Residues involved in binding these entities are shown in stick form.

Of the human proteins predicted to contain the cGST fold, the AlphaFold-predicted structure of the Glutathione S-transferase C-terminal domain-containing protein (GSTCD) is the greatest outlier (Figure 1 and Figure 8). The thioredoxin domain has a region containing a two-stranded β-sheet (β1b + β1c) inserted between strand β1 and helix α2. Following strand β2, a loop and another beta strand (β2b) that forms part of the classic NTD beta sheet is inserted. While the NTD contains the *cis*-proline conserved in cGSTs, the G-site appears to be degenerate; superposition of the GSTCD model with cGST/GSH complexes show clashes with GSH (data not shown). Consistent with this is a lack of detectable GST activity [40]. With the exception of a short helix corresponding to helix α4, the rest of the CTD is rotated approximately 90° with respect to its usual position relative to the NTD. Between helices α5 and α6, a 93-residue helical bundle including a likely disordered 31 residue loop is inserted. Following helix α8 is another extended loop and, finally, the methyltransferase domain. A DALI search of the PDB using human GSTCD as a template yields the methyltransferase domain of *Anabaena variabilis* Hen1-C (PDB ID 3JWH) [41]. Hen1-C is responsible for methylation (using *S*-adenosyl methionine; SAM) of 2′-OH groups at the 3′ ends of small RNAs. Based on crystal structures, it is trivial to model S-adenosyl methionine in GSTCD (Figure 8b,c). A cluster of cysteine residues near the proposed SAM hint at a Zn^2+^ binding site that may be involved in catalysis. The role that the cGST-like components of GSTCD play in function remains unclear, as these parts of the protein do not approach the putative SAM and substrate RNA-binding sites of the methyltransferase domain.

### 3.6. MCS Components

**Figure 9:**
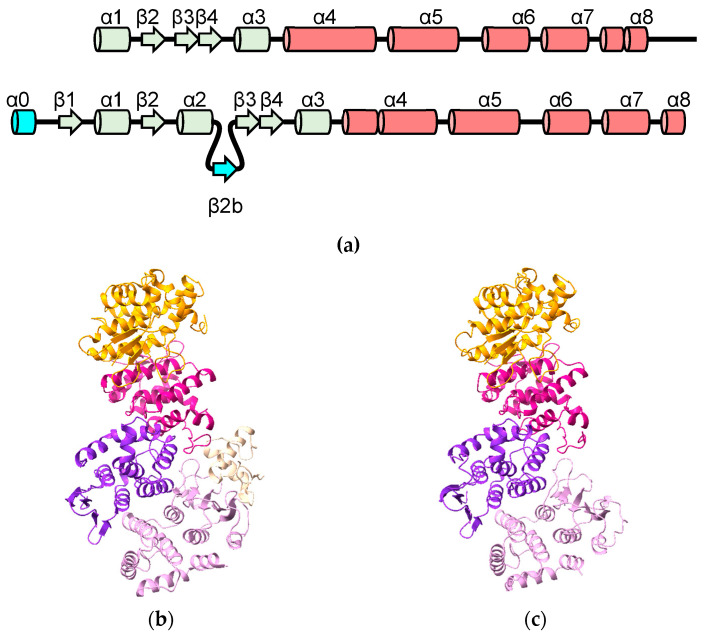
Structures of MCS components: (**a**) topology diagram of EEF1E1 and AIMP2 indicating strands and helices inserted into the cGST domain (α0 and β2b). (**b**) Crystal structure of MARS1 (gold), EEF1E1 (violet-red), EPRS1 (blue-violet) and AIMP2 (plum) complex with DARS1 fragment (peach) (PDB ID 5Y6L). (**c**) AlphaFold prediction of MARS1-EEF1E1-EPRS1-AIMP2 complex with the same color scheme.

Aminoacyl-tRNA synthetases (ARSs) are enzymes that ligate amino acids to their corresponding tRNAs (reviewed by [42]). In eukaryotes, synthetases have been observed to form a complex termed the multisynthetase complex (MSC), an assembly held together by a variety of domains appended to the synthetases as well as structural adapter proteins. The human MSC includes nine ARSs (glutamyl-, prolyl-, isoleucyl-, leucyl-, methionyl-, glutaminyl-, lysyl-, arginyl- and aspartyl-tRNA synthetase). cGST domains appear in four MSC proteins: EPRS1 (glutamyl-prolyl-tRNA synthetase 1), MARS1 (MRS, methionyl-tRNA synthetase 1), AIMP2 (ARS-interacting multifunctional protein 2) and EEF1E1 (eukaryotic translation elongation factor 1 epsilon 1, also known as AIMP3). The cGST domains are essential to the MCS assembly through canonical and non-canonical cGST-dimerization interactions. EEF1E1 has a severely truncated N-terminal domain, missing strand β1 and helix α2. Conversely, AIMP2 has additional secondary structure elements in its N-terminal domain, including an N-terminal α-helix (“α0”) and a strand introduced between β1 and β2 (“β2b”) (Figure 1 and Figure 9a). These novel features were correctly predicted by AlphaFold.

The crystal structures of EPRS1, AIMP2, EEF1E1 and MARS1 with a fragment of aspartyl-tRNA synthetase 1 (DARS1) (PDB ID 5Y6L) reveal canonical heterodimer interactions between AIMP2 and EPRS and between EEF1E1 and MARS1 and a non-canonical interaction between EPRS1 and EEF1E1 (Figure 9b) [19]. AlphaFold models of AIMP2-EPRS1 (MC score 0.93) and EEF1E1 and MARS1 (MC score 0.93) agree with the crystal structures. Despite the interaction between EPRS1 and EEF1E1 being a non-canonical dimerization interaction, AlphaFold correctly predicted the interaction (MC score 0.93). It should be noted that non-canonical complexes of the domains were also predicted, albeit with lower MC scores: EEF1E1-EPRS and EEF1E1-AIMP2 with MC scores 0.89 and 0.86, respectively. Intriguingly, some promiscuity in heterodimer formation is predicted with the four MCS cGST domains. MC scores over 0.9 were observed with GSTP1 with the theta-class GSTs, GSTP1 and CLIC1 (Table S1). The aforementioned CLIC4-AIMP2 complex has an MC of 0.84. Finally, prediction of the complex of the four subunits together was successful (Figure 9c).

Interestingly, EEF1E1, in addition to forming a heterodimer with MARS1, has also been observed to form a homodimer in an X-ray structure (PDB ID 2UZ8) [43]. The MC scores of the predicted complexes are 0.93 and 0.89, respectively.

### 3.7. X EEF1 Components

Eukaryotic elongation factor 1A (eEF1A) binds aminoacyl-tRNAs and delivers them to the ribosome A-site in a GTP-dependent manner. If the correct codon–anticodon interaction occurs, GTP hydrolysis is triggered at eEF1A. GDP-bound eEF1A is then released from the A-site. Translation-elongation factor complex eEF1B facilitates GDP/GTP exchange on eEF1A [44]. Like the MCS, eEF1B is formed from multiple subunits: eEF1Bα (also called elongation factor 1-beta; EF1B), eEF1Bβ (also called elongation factor 1-delta; EF1D) and eEF1Bγ (also known as EEF1G). Additionally, VARS1 interacts with eEF1 to form a “heavy” complex (eEF1H) [45]. Weak GST activity has been found in the rice EEF1G homolog [46].

**Figure 10:**
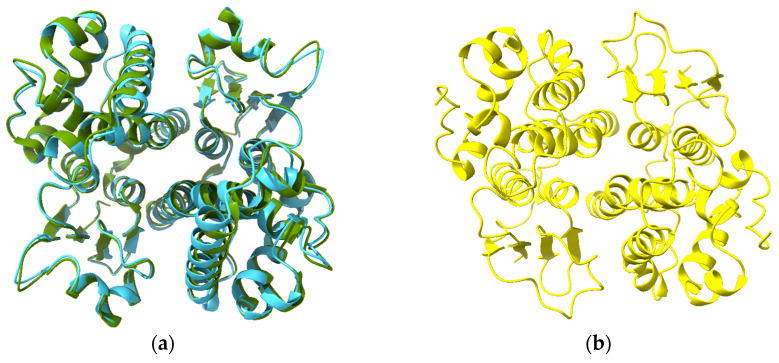
Structures of cGST domains of EEF1 components: (**a**) dimer of the N-terminal domain of EEF1G (crystal structure PDB 5JPO, olive green; predicted structure sky blue); (**b**) predicted structure of VARS1 dimer cGST domain (residues 1–213).

Two components of EEF1 contain (or are predicted to contain) cGST domains: EEF1G and VARS1 (Figure 1). EEF1G is a two-domain protein. Unpublished crystal structures of the human EEF1G N-terminal domain with eEF1Bα (EF1B) (PDB ID 5DQS) and EEF1Bα (EF1D) (PDB ID 5JPO) reveal classic cGST homodimers (Figure 10a). The C-terminal domain of EEF1G was determined by NMR and consists of a five stranded anti-parallel β-sheet surrounded by α-helices [47]. There are no experimental structural data for VARS1. However, the structure predicted by AlphaFold has a cGST-domain at its N-terminus (residues 1 to 213) (Figure 10b). The VARS1 cGST domain is not predicted to contain a residue equivalent to the catalytic residues of catalytically active GSTs and therefore appears unlikely to have enzymatic activity.

Reconstitution of the rabbit eEF1H complex in vitro showed that the cGST domain of VARS1 interacts with eEF1Bβ [48]. The existence of the cGST domains of EEF1G and VARS1 prompts consideration of the possibilities for homo- and heterodimer formation in EEF1H complex formation. Human EEF1G is observed to form classic cGST homodimers in available crystal structures (Figure 10a). However, no experimental structures exist for the VARS1 cGST domain. Pairwise predictions support both homo- and heterodimer formation of EEF1G and VARS1 cGST domains (Table S1). Interestingly, the EEF1G/VARS1 heterodimer gives a higher MC score (0.91) than the VARS1 homodimer (0.84; Figure 10b). Nevertheless, a VARS1 cGST-domain homodimer could be the basis for the reported presence of two copies of VARS1 in the eEF1H complex [49].

## 4. Discussion

The recent advancements in protein structure prediction are leading a revolution in structural biology. While such advances pose obvious opportunities to accelerate research, it is important to determine the strengths and limitations of such tools. The algorithms described and used in this study do not predict the binding of co-factors, metal ions or post-translational modifications. There are also biases associated with the structures used to train the AI, which naturally represent a small fraction of all protein structures. In this study, a bias appears to manifest in the predicted structure of GDAP1, which resembles an archetypal cGST domain more than the available GDAP1 crystal structures. Nevertheless, high-quality predictions can inform experimental strategies. For enzymes such as GSTs, predicted structures could be used (for example) to identify active-site residues for mutagenesis and kinetics studies investigating catalytic mechanism or enzyme–substrate interactions. Where proteins act as adapters for protein–protein interactions, models can be used to identify likely binding interfaces that can again inform mutagenesis studies. For structure determination, the models predict disordered regions likely to inhibit crystallization. Constructs for expression could therefore be designed that truncate or omit such regions.

Based on available experimental data, predicted structures of human cGST-domain-containing dimers appear to be largely correct. Again, one exception is GDAP1: the mode of dimerization is unlike any other known cGST-domain-containing dimer and was not correctly predicted. Nevertheless, the successes found here compare favorably with recent benchmarking studies. Yin and co-workers [50] tested AlphaFold against a set of 152 diverse heterodimer complexes and reported near-native structure predictions for 43% of models. Again, success rates will be influenced by the training data and, therefore, some classes of dimer will be more accurately predicted than others.

Comparisons of predictions of heterodimers with biochemical data yields important lessons. Biochemical detection of heterodimers of mu- and pi-class GSTs [24] support the possibilities presented by predictions of heterodimers between classes of cGSTs (Table S1). As noted above, the propensity for heterodimer formation in alpha-class GSTs is not reflected in MC or ipTM scores. This suggests that these confidence scores should be interpreted as indicating that an interaction is feasible but not necessarily thermodynamically favorable. Multimer formation in vivo will depend on expression levels as well as thermodynamic stability.

## Figures and Tables

**Table 2:** Comparison of predicted structures of human cGST-domain-containing proteins with crystal structures.

Protein	PDB ID	Resolution (Å)	RMSD (Å) ^1^	RMSD (Å) ^1^
GSTA1	1K3Y	1.3	0.353 (220)	0.490 (221)
GSTA2	2VCT	2.1	0.431 (220)	0.459 (221)
GSTA3	2VCV	1.8	0.414 (219)	0.414 (219)
GSTA4	3IK7	1.97	0.425 (218)	0.550 (220)
GSTM1	7BEU	1.59	0.484 (215)	0.551 (218)
GSTM2 ^2^	2C4J	1.35	0.356 (214)	0.438 (217)
GSTM3	3GTU	2.8	0.533 (210)	1.682 (224)
GSTM4	4GTU	3.3	0.586 (216)	0.611 (217)
GSTO1	5YVN	1.33	0.540 (229)	1.082 (238)
GSTO2 ^3^	3Q18	1.70	0.374 (235)	0.412 (236)
GSTP1	5J41	1.19	0.251 (208)	0.251 (208)
GSTT1	2C3N	1.5	0.393 (239)	0.393 (239)
GSTT2	4MPF	2.10	0.302 (243)	0.385 (244)
GSTT2B	4MPC	1.95	0.189 (243)	0.189 (243)
GSTZ1	1FW1	1.90	0.301 (207)	0.334 (208)
HPGDS	7JR8	1.13	0.536 (196)	0.609 (199)
PTGES2 ^4^	1Z9H	2.60	0.490 (266)	0.794 (274)
GDAP1	7AIA	2.2	0.721 (206)	7.511 (259)
CLIC1	1K0M	1.4	0.559 (220)	2.180 (236)
	1RK4 ^5^	1.79	0.569 (165)	7.472 (213)
CLIC2	2R4V	1.85	0.573 (211)	1.962 (226)
CLIC3	3KJY	1.95	0.488 (211)	0.948 (217)
CLIC4	2D2Z	2.20	0.521 (221)	0.828 (229)
CLIC5	6Y2H	2.15	0.518 (210)	1.291 (223)
EEF1E1	2UZ8	2.00	0.677 (154)	1.747 (164)
EEF1G	5JPO	2.00	0.322 (214)	0.322 (214)
AIMP2	5A5H	2.32	0.473 (187)	1.914 (209)
EPRS1 ^6^	5A1N	2.1	0.612 (166)	0.737 (167)
MARS1	4BVX	1.60	0.652 (197)	0.829 (203)

^1^ RMSD values calculated in ChimeraX. The number of Cα atoms used for comparison is provided in parentheses. The first column gives the pruned set of residues that provide the best fit. The second provides RMSD over all matching residues. ^2^ T210S mutant. ^3^ C80S, C121S, C136S, C140S, C170S, C214S mutant. ^4^ From *Macaca fascicularis*. ^5^ Oxidized form. ^6^ S156D mutant.

## Data Availability

Available in Table S1.

## Supplementary Materials

The following supporting information can be downloaded at: https://www.mdpi.com/article/10.3390/biom13081240/s1, Table S1: Model confidence, ipTM and pTM scores for pairwise predictions of cGST-domain-containing proteins. Where experimental structure data are available, pairs are highlighted in red. Figure S1: AlphaFold predictions of GST-domain-containing proteins in the human genome shown in Cartoon form. Structures are colored by pLDDT (key at bottom right). Only the GST domains are shown, except for GSTCD where the whole protein is shown. Figure S2: Phylogram of 39 GST-domain sequences.
