# Supplementary material for: Hidden Glutathione Transferases in the Human Genome
Source: Biomolecules. 2023 Aug 12;13(8):1240. doi: 10.3390/biom13081240 (PMC10452860; doi:10.3390/biom13081240)
Supplement: Supplementary file 1 [file biomolecules-13-01240-s001.zip › Figure_S1.pdf]

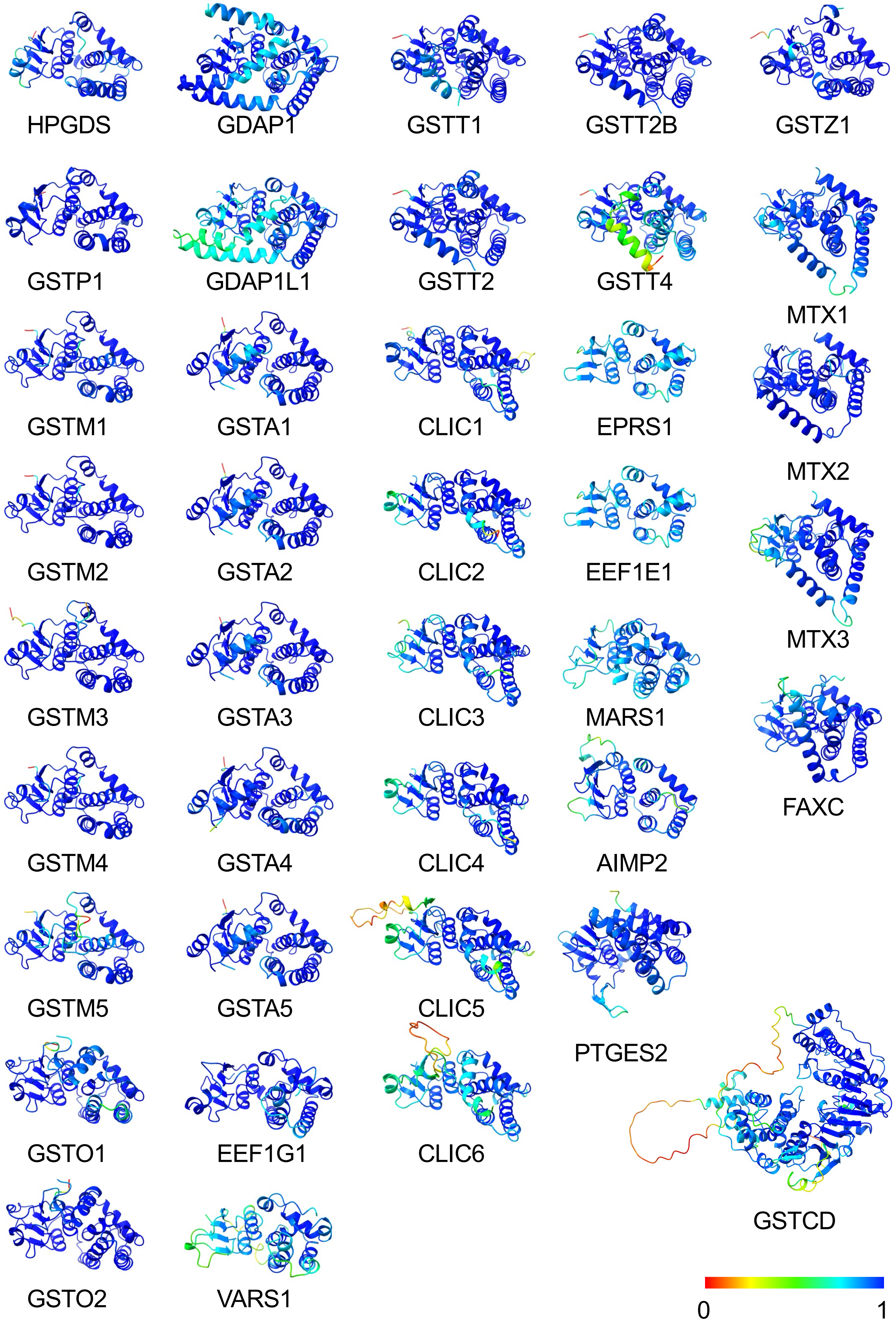


**Figure S1**. AlphaFold predictions of GST domain-containing proteins in the human genome shown in Cartoon form. Structures are colored by pLDDT (key at bottom right). Only the GST-domains are shown, except for GSTCD where the whole protein is shown.
