# Supplementary figures and images for: Hidden Glutathione Transferases in the Human Genome
Source: Biomolecules. 2023 Aug 12;13(8):1240. doi: 10.3390/biom13081240 (PMC10452860; doi:10.3390/biom13081240)

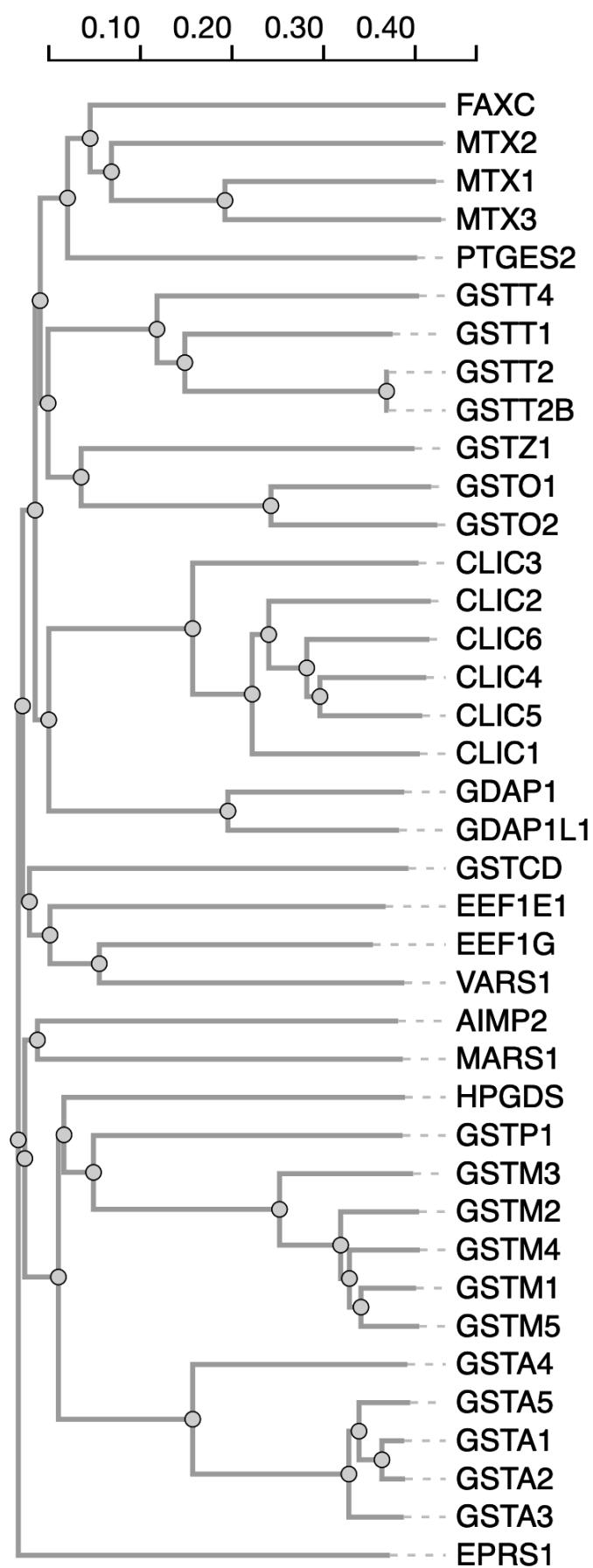

**Figure S2.** Phylogram of 39 GST-domain sequences.

Supplement: Supplementary file 1 [file biomolecules-13-01240-s001.zip › Figure_S2.pdf]
